# Self–Training With Quantile Errors for Multivariate Missing Data Imputation for Regression Problems in Electronic Medical Records: Algorithm Development Study

**DOI:** 10.2196/30824

**Published:** 2021-10-13

**Authors:** Hansle Gwon, Imjin Ahn, Yunha Kim, Hee Jun Kang, Hyeram Seo, Ha Na Cho, Heejung Choi, Tae Joon Jun, Young-Hak Kim

**Affiliations:** 1 Department of Medical Science Asan Medical Institute of Convergence Science and Technology Seoul Republic of Korea; 2 Asan Medical Center University of Ulsan College of Medicine Seoul Republic of Korea; 3 Division of Cardiology, Department of Internal Medicine Asan Medical Center University of Ulsan College of Medicine Seoul Republic of Korea; 4 Big Data Research Center Asan Institute for Life Sciences Asan Medical Center Seoul Republic of Korea

**Keywords:** self-training, artificial intelligence, electronic medical records, imputation

## Abstract

**Background:**

When using machine learning in the real world, the missing value problem is the first problem encountered. Methods to impute this missing value include statistical methods such as mean, expectation-maximization, and multiple imputations by chained equations (MICE) as well as machine learning methods such as multilayer perceptron, k-nearest neighbor, and decision tree.

**Objective:**

The objective of this study was to impute numeric medical data such as physical data and laboratory data. We aimed to effectively impute data using a progressive method called self-training in the medical field where training data are scarce.

**Methods:**

In this paper, we propose a self-training method that gradually increases the available data. Models trained with complete data predict the missing values in incomplete data. Among the incomplete data, the data in which the missing value is validly predicted are incorporated into the complete data. Using the predicted value as the actual value is called pseudolabeling. This process is repeated until the condition is satisfied. The most important part of this process is how to evaluate the accuracy of pseudolabels. They can be evaluated by observing the effect of the pseudolabeled data on the performance of the model.

**Results:**

In self-training using random forest (RF), mean squared error was up to 12% lower than pure RF, and the Pearson correlation coefficient was 0.1% higher. This difference was confirmed statistically. In the Friedman test performed on MICE and RF, self-training showed a *P* value between .003 and .02. A Wilcoxon signed-rank test performed on the mean imputation showed the lowest possible *P* value, 3.05e-5, in all situations.

**Conclusions:**

Self-training showed significant results in comparing the predicted values and actual values, but it needs to be verified in an actual machine learning system. And self-training has the potential to improve performance according to the pseudolabel evaluation method, which will be the main subject of our future research.

## Introduction

### Background

When trying to use data in machine learning or statistical analysis, the missing value problem is one of the most common challenges. A missing value is caused by situations such as a malfunction of the inspection machine, incorrect inspection, or human error. It can also happen when converting data for analysis purposes. Missing values reduce the number of data points available and adversely affect the analysis results. In the medical field, inaccurate analysis is fatal as it can lead to a misdiagnosis. The best way to deal with this problem is to fill in missing values with the actual values. However, filling up medical data with the real values may require expensive retesting or assistance from a professional medical practitioner, which is extremely cumbersome and costly. Also, it may be impossible to fill in missing values due to patient privacy issues. For this reason, many studies on the imputation of missing values have been conducted.

The most naive way to fill in the missing value is to fill it with an appropriate value such as zero or the average. Mean imputation is one of the most frequently used methods because it is simple. In other cases, the median and mode can be used as substitutes instead of using the mean. These methods have the disadvantages of increasing errors and introducing bias in datasets with a high number of missing data points. In addition to these simple methods, attempts have been made to resolve the missing value problem statistically, represented by expectation-maximization (EM) [1] and multiple imputations by chained equations (MICE) [2]. EM finds the local minimum and does not guarantee that the value found is the global maximum. Additionally, since it is a single imputation replacing only one value, the accuracy may be degraded when the missing rate of the data is large. The multiple imputation (MI) [3] method compensates for this shortcoming of single imputation. In MI, several imputed values are statistically analyzed and used. MI works under missing at random (MAR) [4] conditions. MICE is one of the MI algorithms. MICE performs statistical modeling by creating several imputation sets of missing values through simulation and derives values by averaging the generated imputation sets. Like any other MI algorithm, MICE operates under the assumption of MAR, and execution under the assumption that it is not MAR can lead to biased results.

As research on machine learning becomes more active, machine learning algorithms such as multilayer perceptron (MLP) [5,6], k-nearest neighbor (KNN) [7,8], and decision tree (DT) [9-11] have been used for imputation as alternatives to statistical methods. Recently, generative models such as generative adversarial networks [12,13] have been applied to missing value problems as they have shown significant performance in several fields. Jerez et al [14] compared statistical programs and machine learning methods to replace missing values in breast cancer datasets. In their study, mean, hot deck [15,16], SAS [17], Amelia [18], and MICE were used as the statistical methods, and MLP, KNN, and self-organizing map [19] were used as the machine learning methods.

### Objective

The objective of this study was to impute numeric data such as physical data and laboratory data. Laboratory data and physiological data are valuable data that directly represent the patient's health condition, and these continuous values are usually harder to predict than discrete ones, making them worth studying. On the other hand, discrete data, such as the diagnosis reached by the physician, may not be appropriate to impute as an external factor. Since the patient does not undergo all of the tests, just the necessary tests, there is always a missing value in the electronic medical record (EMR) data. Unlike the universal tests performed on many patients, some tests are performed on only some patients. Features corresponding to these special tests are suitable for imputation because there are many missing parts and they have a high potential advantage.

EMR data have characteristics that distinguish it from other data, and our objectives are subdivided according to these characteristics [20-23]. The most distinctive feature of EMR is that there is a difference in the missing rate between features, and this difference in missing rates is an important consideration for imputation. Therefore, our first detailed objective was to impute rare features using general features.

Another characteristic of EMR is that there are far more incomplete (or unlabeled) data than complete (or labeled) data. In fact, although most of the data have this property, medical data are much more lethal because the process of obtaining labels is expensive and cumbersome. Existing methods can be vulnerable in these circumstances, and the second aim of our progressive method was to ameliorate these vulnerabilities.

To overcome this vulnerability, we adopted self-training, a progressive method, in which self-training performs 2 processes repeatedly: self-learning and pseudolabeling. Self-training repeats the 2 processes using a complete dataset called a teacher and an incomplete dataset called a student. First, the teacher model learns the teacher dataset, and then the teacher model predicts the missing values of the student data. This step is called pseudolabeling. Second, the valid pseudolabeled student data are converted to teacher data. In the next iteration, the same process is repeated with this new teacher, which is called self-learning.

The most important part of the self-training process is how to evaluate the validity of the pseudolabel. However, the actual value is unknown, which is why we cannot directly evaluate its validity; thus, a new evaluation method is necessary. In the classification problem, probability is used as an indicator, and a simple example can be found through binary classification. Suppose that a model trained with labeled data predicts that the test set has a 96% probability of being negative and a 4% probability of being positive. This result can be interpreted as meaning the model is confident that the predicted class is negative. Conversely, if the model predicted the test set has a 55% probability of being negative and a 45% probability of being positive, the interpretation is unclear. In the former case, it can be said that the test set belongs to the negative class, but in the latter case, it is difficult to determine whether it is positive or negative. In the case of self-training, the test set in the first case is labeled as a negative class and transferred to the teacher. Unfortunately, there is no such intuitive judgment factor in the regression problem. Therefore, most studies related to self-training are conducted mainly using classifications like image net challenges [24]. Our final objective was to apply this self-training to the regression problem.

## Methods

**Figure 1:**
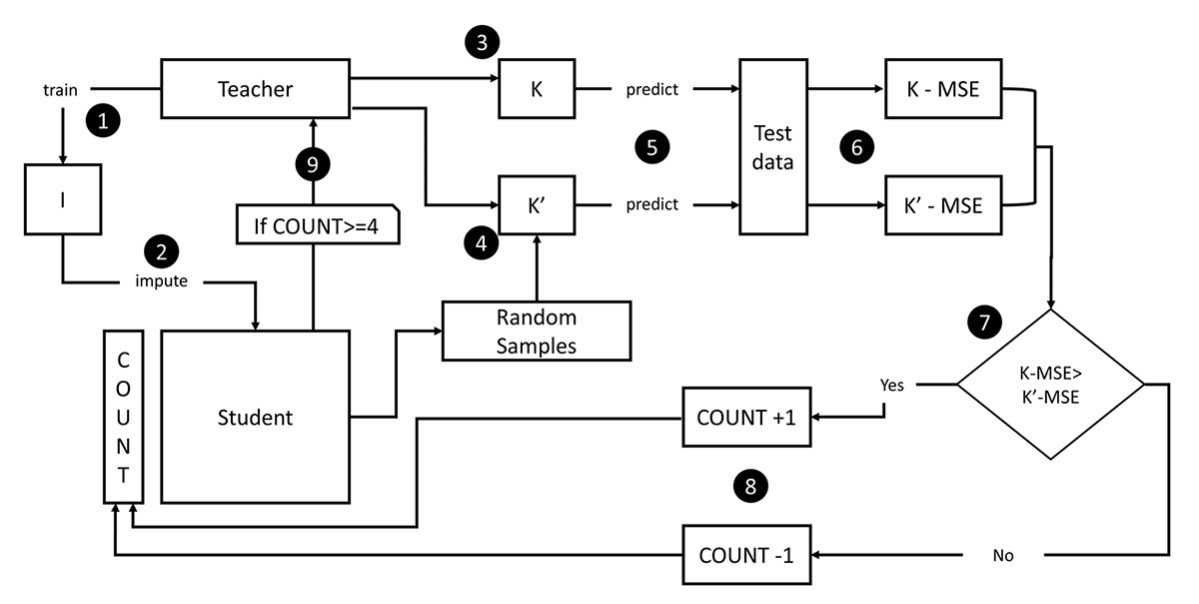
Architecture of the self-training process. MSE: mean squared error.

In this section, we present an approach to semisupervised learning for continuous EMR imputation. Our approach is based on a self-training paradigm, and we named it SQMI-R. Figure 1 shows a full overview of the proposed method. The numbers in Figure 1 represent the sequence of the process, and each sequence is explained in the following subsections. The Self-Training Regression Imputation section describes number 1 to number 6 of the process, and the Sampling Strategy section considers number 4 in more detail. The Evaluation Metrics section analyzes step number 6 in more detail, and the Multiple Imputation section describes steps 6 through 9.

### Self-Training Regression Imputation

**Figure 2:**
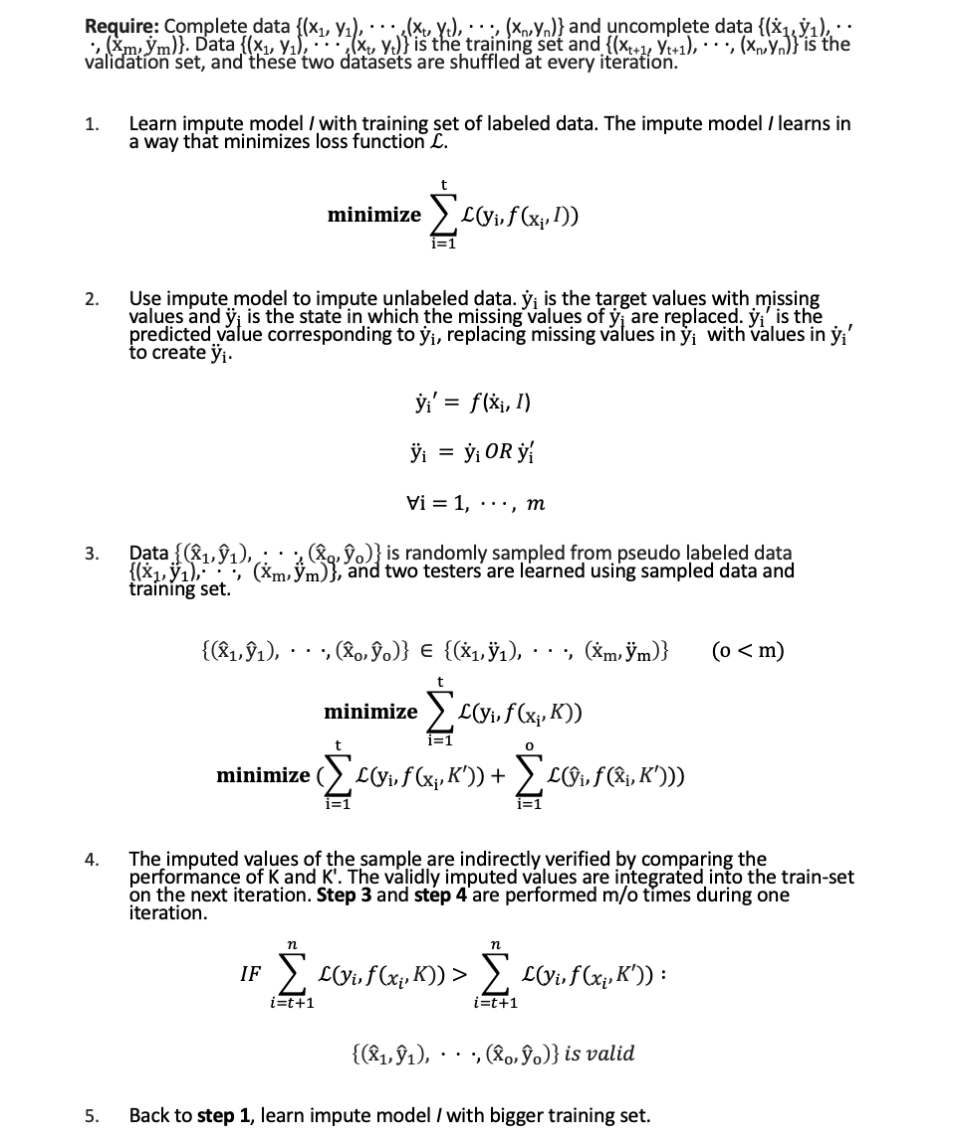
Validation of the imputed samples.

SQMI-R uses 3 models to impute continuous values: I, K, and K’. First, we pseudolabel the missing values of the student dataset with the imputation model I trained with the teacher dataset. Then, 2 test models K and K’, called the tester, train 2 nearly similar datasets. K learns only from the teacher, and K’ learns data from both the teacher and samples of the pseudolabeled student. In general, in the case of adding data to an existing machine learning system, it can be said that if the added data are valid, the performance of the model improves; otherwise, the performance decreases. We used the properties shown in Figure 2 to validate the imputed samples.

This process is based on the assumption that valid data improve the performance of the model. Based on these assumptions, the imputed samples are added to the existing data and verified based on performance improvements. The added amount of data is too small compared to the existing data and has a minimal performance impact. Both test models should be able to detect even these small effects, and we used KNN as a tester to satisfy this requirement. KNN is useful for detecting small differences in the data as it always produces the same results for the same data. The special behavior of the KNN algorithm makes it possible to always derive the same value. KNN estimates labels from the average of the surrounding k data without a learning process, which always produces the same results for the same data. Algorithms such as MLP and RF require a learning process, and randomness intervenes, resulting in different results for the same data. In this case, it is difficult to define whether the difference in results is due to differences in the data or randomness of the learning. Although KNN is not performing as well as these machine learning models, the purpose of the test model is to compare and verify the data, not accurately predict it. Moreover, KNN has few parameters to process and is intuitive to use. The loss function ℒ also has to be chosen carefully for each purpose. Since there was no specific purpose in this study, mean squared errors (MSEs) that could be used for all continuous value problems were adopted. If the purpose is to learn a classifier, the process can be performed by using cross-entropy as a loss function or by maximizing metrics such as area under the curve or F1 scores.

### Sampling Strategy

The most reliable way to examine pseudolabeled data is to examine them one by one. Nevertheless, the reason for testing multiple samples instead of testing them one by one is related to the characteristics of the KNN. KNN is calculated based on the k-nearest data. Consider the case of testing only one sample x. There may be cases where this x is far from all of the test data. In this case, adding x to K’ does not affect the test result. When these cases increase, useless calculations increase, and self-training does not work smoothly. If enough samples are used at once, the validity of the pseudolabel will affect the performance of the model, and accordingly, the validity of the sample can be verified. In this experiment, we adopted 50 samples. After several tests, we selected the 50 that seemed to be the most appropriate in terms of the trade-off between the performance and time. The number of samples is an important parameter. In general, as the number of samples increases, the speed will increase, whereas the performance decreases. Contrarily, if the number of samples decreases while the performance improves, the time efficiency gets worse. Thus, the number of samples should be chosen appropriately in the trade-off of the relationship between the time and the performance.

If the number of samples obtained from a single sampling has been determined, how many samplings should be performed during one iteration should be determined. In our strategy, if the number of students is S and the number of samples drawn at one time is N, sampling is done S/N times during one iteration. Such a sampling strategy can, on average, examine all data once during one iteration.

### Evaluation Metrics

It is necessary to think about the evaluation metrics when testing the sample. We imputed multiple features, and the effectiveness of the pseudolabel was evaluated by the MSE of the actual and predicted values. This MSE is affected by the distribution of the features. A feature with low density (ie, a wide distribution of data) has a structurally higher MSE in prediction than a feature with a high density and a narrow distribution. Models K and K’ test samples using the average MSE of all features. If the density of the data determines the MSE, self-training will work differently than we expected. In other words, self-training will only work around features with widely distributed data to reduce the overall error. This is because reducing errors from data with a low density is more advantageous in reducing the total errors than reducing the errors from data with a high density. Due to the high data density, neglected features may be less improved or worsened during the self-training process.

**Figure 3:**
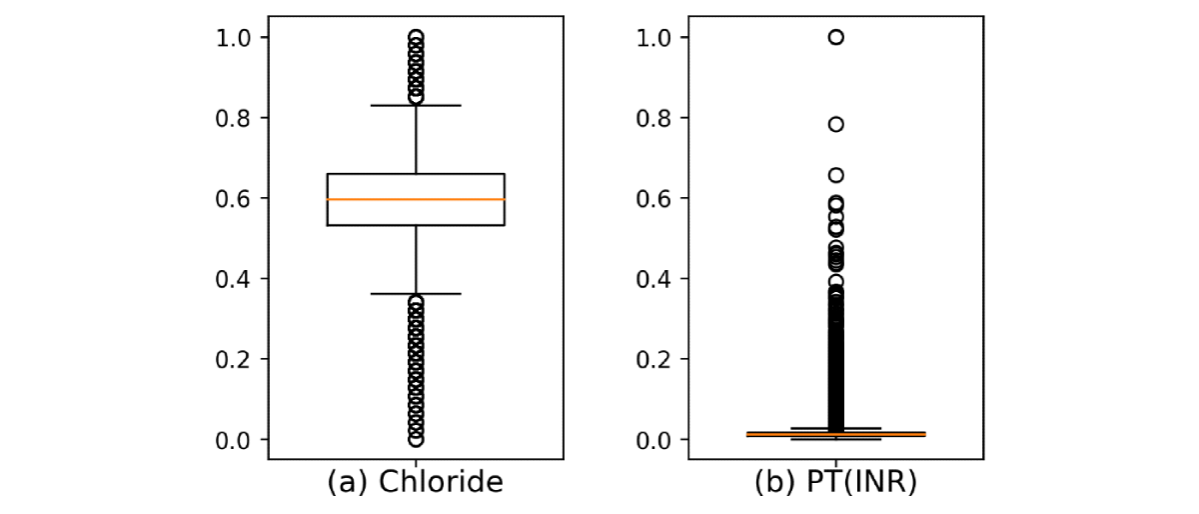
(A) Chloride has a low density, and (B) PT(INR) has a high density. PT(INR): prothrombin time(international normalized ratio).

We can confirm this with practical medical records. The collection of data and data preparation received Asan Medical Center and Ulsan University Hospital institutional review board approval with waived informed consent (AMCCV 2016-26 ver2.1) [25]. Figure 3 shows a boxplot of 2 features — chloride and PT(INR). Note that all features are min-max scaled in Figure 3. The data from PT(INR) are distributed over a small area. Consider the case of predicting a value in the box of PT(INR). Since the box itself is small, the prediction error is small, and the result looks accurate. On the contrary, chloride is distributed over a large area, and even if the predicted value is in the box, the error will be relatively large. In this case, chloride has a greater impact on the overall error. As a result, the self-training process works toward improving chloride even further, even if it worsens PT(INR).

Medical data have large differences in the data distributions between features, and self-training is vulnerable to such characteristics. The evaluation of the effectiveness of the pseudolabel is based on the average MSE of the features. However, evenly reducing the MSE of all features is a way to make a better dataset. Therefore, it is necessary to correct the effect of the distribution. In this study, we presented a correction method using quantiles. The distribution of the data is estimated using the interquartile range (IQR). IQR is the difference between the third and first quartiles. If the data density is high, the difference between the third and first quartiles will be small. If the density is small, the difference between the third and first quartiles will be large. Let the third quartile of i-th feature be *q_i_^3^* and the first quartile be *q_i_^3^*, then the IQR of the i-th feature can be defined as follows:

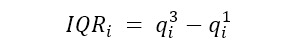



IQRi is divided by MSE, which is inversely proportional to the distribution of the data. We named it Q-MSE, and the definition is as follows:

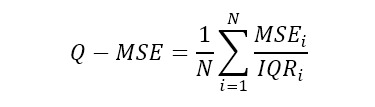



In the case of using Q-MSE, if the Q-MSE of K’ is smaller than the Q-MSE of K, it is assumed that the imputed value is valid.

### Multiple Imputation

The method of evaluating multiple samples at once has a vulnerability. Assume that data X1 are effectively pseudolabeled. However, if X1 was sampled with invalid data, it will degrade the performance of K’ in the test. If this happens, even though X1 should be a teacher, it will remain in the student due to bad luck. This time, we can think of a case in which invalid data X2 are sampled with valid data. X2 and valid samples will improve the performance of the model. In this case, X2 is not valid, but it becomes a teacher. Since X2 is invalid data, if it becomes a teacher, the performance of the algorithm degrades. We present ways to prevent this irrationality.

**Figure 4:**
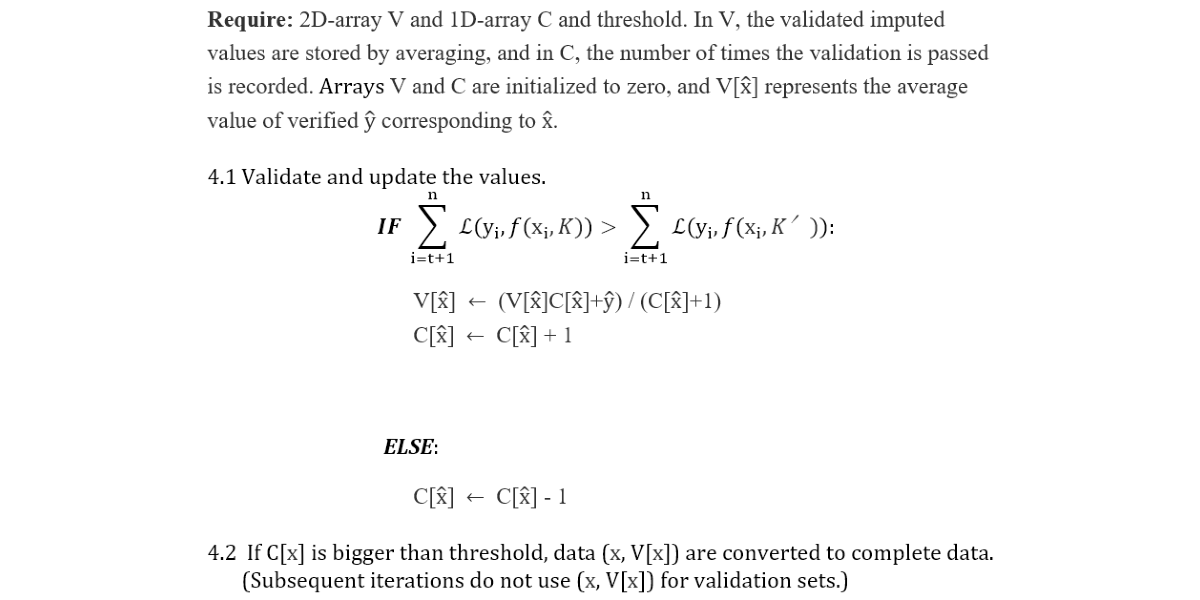
Detailed steps within step 4.

In the proposed method, students get a new imputed value at each iteration, and if the test passes, this imputed value is stored. In the next iteration, it gets a new value and is tested again. If the test passes again, the stored value is updated by averaging the current value and the stored value. To manage this stored value, we count the number of passes. If the test passes, we add 1 to the count of the data. If it fails the test, the count decreases by 1. Data that count as greater than the threshold become teachers by replacing the missing value with this stored value. Data with a zero count return the stored value to zero. Groups containing X1 already have valid data, which is likely to improve the performance of the model. Thus, X1 has a relatively high probability of passing the test while it belongs to several samples, and it will go to the teacher by filling the count with a threshold with a relatively high probability. Invalid data have a high probability of dropping from the test, deducting 1 point from the counter. In this way, it is possible to avoid making choices by chance. In addition, the values verified from various test data are integrated to make the performance stable. According to this strategy, step 4 from the Self-Training Regression Imputation section is divided into the detailed steps seen in Figure 4.

Setting the threshold of counts is a trade-off between performance and the time required. If you set the threshold higher, you will have to perform more tests and filter more verified data. However, this requires too many iterations to make it into the labeled data. In some cases, the performance can become worse by reducing the number of incorporated data. On the other hand, lowering the threshold shortens the time and increases the amount of data transferred to the labeled set, but it does not guarantee the quality of the data. The lower the threshold, the greater the influence of luck. As a result of conducting several tests, we found that approximately 4 counts could obtain appropriately verified pseudolabeled data with optimized periods of time. Thus, in this experiment, we use 4 for the threshold of the counts.

**Figure 5:**
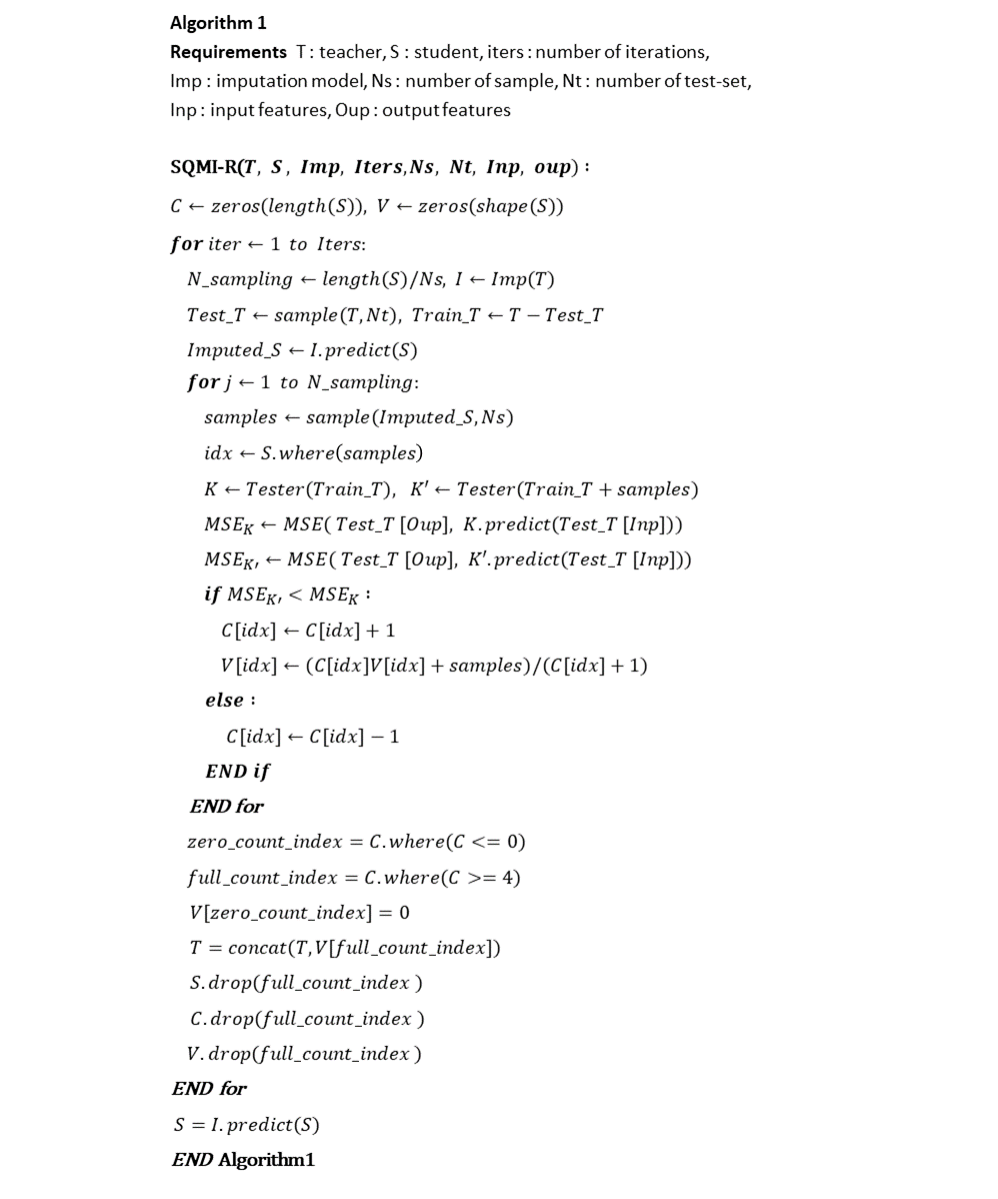
Pseudocode of the SQMI-R.

Algorithm 1 in Figure 5 shows the pseudocode of the SQMI-R. Model(data) means that the model is trained with data. For example, Tester(data) means that a Tester model was trained using data. I.predict(data) means impute by predicting the missing value of the data with the imputing model I. Data[index] represents values corresponding to the index of the data.

## Results

### Dataset

We validated our method with data from CardioNet [25], a real-world EMR. The demographic information from CardioNet appears in Table 1, and we selected 10,000 of the data points as the teacher data and 50,000 of the data points as student data. A teacher dataset with 10,000 data points represents complete data without missing values, and the 50,000 students contain missing values. The actual values in the student dataset are unknown and cannot be evaluated for imputation. For evaluation, we used some of the known values from the students as fake missing. A total of 93 features such as physical information, laboratory tests, and the results of echocardiography were used, and 77 of these were used as inputs to impute the missing values of the remaining 16 targets: chloride, alkaline phosphatase, protein, total CO_2_, glucose, uric acid, blood urea nitrogen, electronic absolute neutrophil count, phosphorus, prothrombin time (PT-INR, PT-%, PT-sec), systolic blood pressure, diastolic blood pressure, pulse rate, respiratory rate. The missing rate of the features varied from 0.06% to 85.9%. Table 2 shows the missing rate for each feature. The patterns of missing data include MAR, missing completely at random, and missing not at random [4]. The decision to perform a medical test is usually determined by other observed information. Therefore, the missing laboratory data are related to the observed values, and the pattern is MAR.

**Table 1:** Demographic information from the CardioNet electronic medical record.

Variables			Asan Medical Center (N=572,811)
**Gender, n**			
	Female	257,160	
	Male	315,651	
Age (years), mean (SD)			56.32 (14.72)
Systolic blood pressure^a^ (mm Hg), mean (SD)			123.06 (12.61)
Diastolic blood pressure^a^ (mm Hg), mean (SD)			74.29 (7.94)
BMI^b^ (kg/m^2^), mean (SD)			24.11 (3.50)
**CV/CS^c,d^ encounter, n**			
	0	250,160	
	1	68,037	
	2	78,406	
	≥3	174,560	
Echocardiography, n (%)			428,004 (74.71)
Pulmonary function, n (%)			265,817 (46.40)
Thallium SPECT^e^, n (%)			156,615 (27.34)
Treadmill, n (%)			68,203 (11.90)
CT^f^, n (%)			79,064 (13.80)
Holter monitoring, n (%)			46,636 (8.14)
6-minute walk test, n (%)			8871 (1.54)
Cardiac rehabilitation, n (%)			1990 (0.34)
Pediatric echocardiography, n (%)			1720 (0.30)

^a^N=461,693.

^b^N=457,621.

^c^CV/CS: Cardiology or Cardiothoracic Surgery Department.

^d^571,163 total visits.

^e^SPECT: single photon emission computed tomography.

^f^CT: computed tomography.

**Table 2:** Missing rate and ratio of errors (obtained by dividing the result of 20 iterations by that of 0 iterations).

Feature	Missing rate, %	Normal-MSE^a^	Q-MSE
Chloride	17.60	0.866	0.867
AP^b^	1.00	0.917	0.910
Protein	0.06	0.839	0.837
Total CO_2_	28.75	0.906	0.902
Glucose	71.38	0.844	0.844
Uric acid	53.11	0.872	0.865
BUN^c^	59.60	0.713	0.709
E_ANC^d^	74.80	1.007	0.993
PT(INR)^e^	0.10	0.962	0.968
PT (%)	17.71	0.988	0.982
Phosphorus	0.19	0.886	0.884
PT (sec)	27.98	0.950	0.954
SBP^f^	71.38	0.983	0.975
DBP^g^	77.68	0.977	0.977
PR^h^	59.00	0.994	0.993
RR^i^	85.86	1.005	0.993

^a^MSE: mean squared error.

^b^AP: alkaline phosphatase.

^c^BUN: blood urea nitrogen.

^d^E_ANC: electronic absolute neutrophil count.

^e^PT(INR): prothrombin time(international normalized ratio).

^f^SBP: systolic blood pressure.

^g^DBP: diastolic blood pressure.

^h^PR: pulse rate.

^i^RR: respiratory rate.

### Experiments on the Effects of the Metric

We experimented on the 2 aforementioned metrics (normal-MSE, Q-MSE) to confirm the change of self-training according to the metric. All experiments were conducted based on a situation where the missing rate was 20% and the number of iterations was 20. Table 2 is the result of dividing the result of 20 iterations by that of 0 iterations when each metric is applied. This ratio indicates how the error decreases when the process ends as compared to the starting point. The smaller ratio values indicate better performance, with a value of 1 indicating that the process has no effect and values greater than 1 indicating that the process is adversely affected. This ratio can be influenced by the degree of ease of prediction, data distribution, and missing rate. We assumed that performance could be affected by IQR, and Q-MSE was suggested to compensate. The results are shown in Table 2. Q-MSE increased the error rate in 3 features compared with normal-MSE but decreased it in 11 features. In this experiment, we can confirm that the assumptions we have set are correct and that the method we have presented is also effective. Furthermore, the results of this experiment suggest that properly setting the evaluation metric of the pseudolabel can improve the performance of the imputation.

### Comparison With Existing Methods

The second experiment compared the performance of the existing imputation method and SQMI-R by the missing rate. The self-training iteration was fixed to 20 times, and the metric was normal-MSE. After setting various missing rate situations, we evaluated the performance of the methods in each situation. Then, some of the actual values of the student data missing in the experiments were filled in. Finally, the results of the imputation were evaluated by MSE and the Pearson correlation coefficient.

All experiments were conducted in *Python-3.6.9* environments, and each algorithm was implemented through the *Python* library. We utilized *sklearn-0.23.2* to implement the machine learning models, RF and KNN, and MLP was implemented in *keras-2.24*. The statistical methods, EM and MICE, were implemented through *impyute-0.0.8.* All statistical analyses were performed via *scipy-1.5.2.*

#### Mean Squared Error

**Figure 6:**
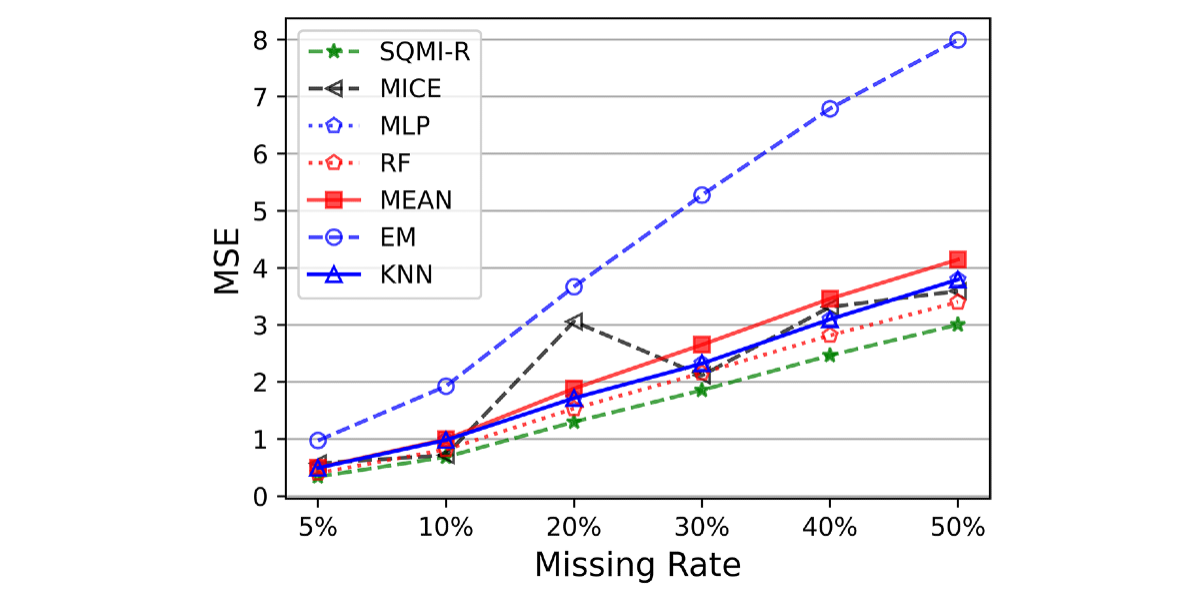
Total errors from the methods in each missing rate stage. EM: expectation-maximization; KNN: k-nearest neighbor; MICE: multiple imputations by chained equations; MLP: multilayer perceptron; MSE: mean squared; RF: random forest.

**Figure 7:**
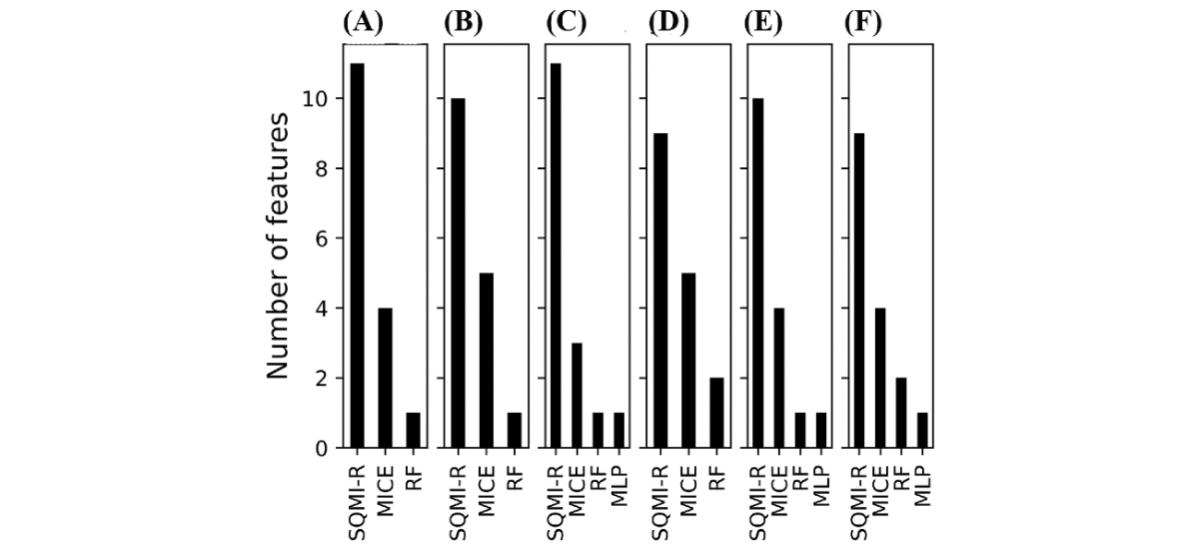
The number of features that the method predicted best in each missing rate stage: (A) 5%, (B) 10%, (C) 20%, (D) 30%, (E) 40%, (F) 50%. MICE: multiple imputations by chained equations; MLP: multilayer perceptron; RF: random forest.

Figure 6 presents the MSE performance of the methods according to the missing rate. As expected, the error increased as the missing rates increased. In the graph, the SQMI-R is the most robust for the increase in the missing rate. Accordingly, the higher the missing rate, the more efficient the SQMI-R. Figure 7 presents the number of features best predicted by the method. Looking at Figure 7, SQMI-R performed better than other methods for most features in all stages. It showed the lowest error in at least 9 and at most 11 features depending on the missing rate. After SQMI-R, MICE had most of the features, followed by RF and MLP.

To more accurately evaluate the differences between the methods, we performed the Friedman test [26] on the most powerful 3 algorithms: RF, MICE, SQMI-R. The Friedman test is a nonparametric test that verifies the significance of differences between N algorithms. The Friedman test is used to detect differences between algorithms in multiple test attempts. Columns (repeated tests attempts) rank rows (algorithms) and analyze these ranks to detect differences between algorithms. If there is a superior algorithm, it will rank high in most columns. The Friedman test requires results for iterative experiments such as cross-validation. However, in this study, both student data and teacher data are defined, making this iterative experiment difficult. Therefore, instead of using the results from iterative experiments, the rankings of multiple features were compared. There is no difference between the algorithms under the null hypothesis. Table 3 presents the results of the Friedman test at each step. The *P* values from the Friedman tests were <.05 in all missing rate situations, and the null hypothesis was rejected, which means that there is a significant effect on the method in all steps. This result demonstrates that SQMI-R is statistically superior to other powerful algorithms, referring to other results that will be presented later.

**Table 3:** Friedman test *P* values for self-training, multiple imputations by chained equations (MICE), and random forest.

Missing rate	*P* value
5%	.003
10%	.005
20%	.003
30%	.02
40%	.007
50%	.02

Additionally, we conducted Wilcoxon signed-rank tests [27] to verify that there are significant differences between mean imputation and the 6 methods. The Wilcoxon signed rank test is a nonparametric test method that determines whether the medians of paired data are the same. The test calculates the difference between paired data and then the signed rank of the difference to obtain the test statistic. In the null hypothesis, the difference between 2 paired data forms a symmetrical distribution around zero. In this study, we used the imputation results for multivariate target features to determine significant differences between each method and mean imputation. Table 4 presents the results of the Wilcoxon signed-rank test at each step. For MLP, the *P* value was larger than .05 at most stages, which means that this method is not significantly different from the mean imputation. Whether KNN and MICE reject the null hypothesis depends on the missing rate, which means that in some cases, there may be no difference from the mean imputation. SQMI-R, RF, and EM showed the smallest possible *P* values at all stages. In Figure 6, EM had a higher error compared to mean imputation, and a small *P* value means that EM is inferior to mean imputation for all features. The single imputation EM is unstable when the missing rate is large, which is consistent with the experimental results. Only RF and SQMI-R are methods superior to mean imputation for all features in all situations. The comparison between these 2 models is meaningful as RF is used as the impute model in SQMI-R. At 50%, as seen in Figure 6, SQMI-R improved by about 12% when compared to RF. These results proved that self-training could improve the performance of the imputation model while preserving the statistical significance.

**Table 4:** *P* values from the Wilcoxon signed-rank test for mean imputation.

Missing rate	MLP^a^	RF^b^	MICE^c^	EM^d^	KNN^e^	SQMI-R
5%	.86	<.001	.005	<.001	.463	<.001
10%	.12	<.001	<.001	<.001	.668	<.001
20%	.07	<.001	.375	<.001	.013	<.001
30%	.001	<.001	<.001	<.001	<.001	<.001
40%	.06	<.001	.252	<.001	<.001	<.001
50%	.06	<.001	<.001	<.001	<.001	<.001

^a^MLP: multilayer perceptron.

^b^RF: random forest.

^c^MICE: multiple imputations by chained equations.

^d^EM: expectation-maximization.

^e^KNN: k-nearest neighbor.

#### Pearson Correlation Coefficient

We calculated the Pearson correlation coefficient to evaluate the imputation data in another way. We experimented with a situation where the missing rates were 10%, 20%, 30%, 40%, and 50%, and 32,302 data points were used. We used 7000 of these as training data, and randomly created missing data for the rest of the data and used them as test data. Subsequently, the Pearson correlation coefficient between the 25,302 imputed test data points and the original data was calculated to represent the correlation between 2 vectors. The Pearson correlation coefficient has a value between +1 and –1, where +1 means a strong positive linear correlation, 0 means no linear correlation, and –1 means a strong negative linear correlation. In this study, the Pearson correlation coefficient was used as an indicator to measure the degree of preservation of the data structures in the imputed data. It is more important to preserve the data structure when replacing the missing values than simply reducing the integrated error.

**Figure 8:**
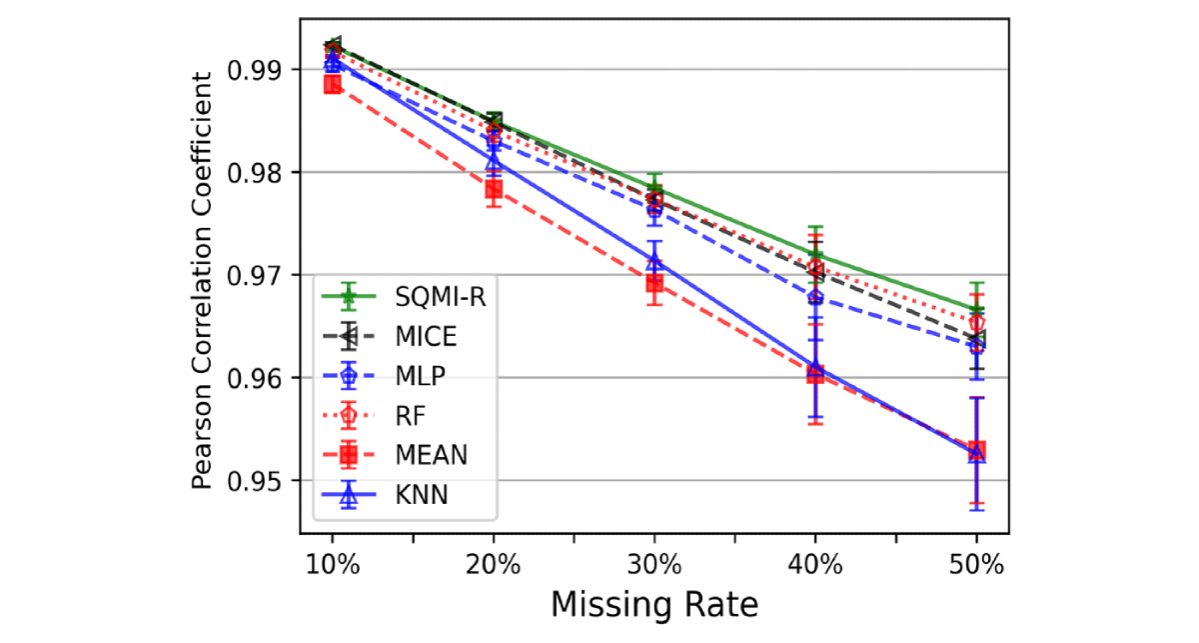
Pearson correlation efficient in various missing rate situations. KNN: k-nearest neighbor; MICE: multiple imputations by chained equations; MLP: multilayer perceptron; RF: random forest.

As shown in Figure 8, the mean and variance of the Pearson correlation coefficients was almost similar to the results of Figure 6. As the missing rate increases, SQMI-R keeps the Pearson correlation coefficient higher than in the other methods. At each point on the graph, the vertical line represents the variance, while the SQMI-R has the lowest variance. This result implies that SQMI-R is most strongly correlated with the original data, which has great significance in terms of data utilization.

## Discussion

In this study, we proposed multiple self-training regression imputation methods. The proposed algorithm used 3 models. We named the complete data set the teacher and the data set with missing values the student. The missing value of students was predicted with imputation model I, and these predicted values were then evaluated with test models K and K’. If this prediction is determined to be valid, the student becomes a teacher. The data remaining as a student until the end were predicted and imputed by the final imputation model. The first experimental result showed that the metric we presented, Q-MSE, works better than normal-MSE. In the second experimental result, it was confirmed that the self-training imputation was statistically significantly superior to the existing statistics and machine learning methods.

Self-training is one independent process, but it is also a process that further enhances existing methods. The relationship between RF and SQMI-R demonstrates this well. Our method can be easily combined with other algorithms as well as RF and is expected to improve these algorithms. The most important thing in this process is the metric. The purpose or aspect of self-training can vary greatly depending on the metric, so the appropriate metric should be used. In this work, we proposed a metric assuming that all target features are continuous, but for general use, we need a metric that can be used when continuous and discrete values are mixed. And our algorithm requires repeated measurements, which are time-consuming. This limitation is one of the challenges that we need to optimize. Furthermore, experiments on whether the proposed imputation is well applied to practical statistical analysis or machine learning problems are also needed. Applying our method to real machine learning problems with complex data will be the main subject of our future research.

## Abbreviations

DT: decision tree
EM: expectation-maximization
EMR: electronic medical record
IQR: interquartile range
KNN: k-nearest neighbor
MAR: missing at random
MI: multiple imputation
MICE: multiple imputations by chained equations
MLP: multilayer perceptron
MSE: mean squared error
